# Musashi2 predicts poor prognosis and invasion in hepatocellular carcinoma by driving epithelial–mesenchymal transition

**DOI:** 10.1111/jcmm.12158

**Published:** 2013-10-31

**Authors:** Lu He, Xinke Zhou, Chen Qu, Lijuan Hu, Yunqiang Tang, Qiong Zhang, Min Liang, Jian Hong

**Affiliations:** a Department of Hepatobiliary Oncology Affiliated Tumour Hospital, Guangzhou Medical UniversityGuangzhou, Guangdong, China; b Cancer Institute, Central South UniversityChangsha, Hunan, China; c Department of Medical Oncology Affiliated Tumor Hospital, Guangzhou Medical UniversityGuangzhou, Guangdong, China; d Department of Pathophysiology Affiliated Tumor Hospital, Guangzhou Medical UniversityGuangzhou, Guangdong, China

**Keywords:** hepatocellular carcinoma, musashi (MSI), invasion, prognosis, biomarker

## Abstract

The high incidence of recurrence and the poor prognosis of hepatocellular carcinoma (HCC) necessitate the discovery of new predictive markers of HCC invasion and prognosis. In this study, we evaluated the expression pattern of two members of a novel oncogene family, Musashi1 (MSI1) and Musashi2 (MSI2) in 40 normal hepatic tissue specimens, 149 HCC specimens and their adjacent non-tumourous tissues. We observed that MSI1 and MSI2 were significantly up-regulated in HCC tissues. High expression levels of MSI1 and MSI2 were detectable in 37.6% (56/149) and 49.0% (73/149) of the HCC specimens, respectively, but were rarely detected in adjacent non-tumourous tissues and were never detected in normal hepatic tissue specimens. Nevertheless, only high expression of MSI2 correlated with poor prognosis. In addition, MSI2 up-regulation correlated with clinicopathological parameters representative of highly invasive HCC. Further study indicated that MSI2 might enhance invasion of HCC by inducing epithelial–mesenchymal transition (EMT). Knockdown of MSI2 significantly decreased the invasion of HCC cells and changed the expression pattern of EMT markers. Moreover, immunohistochemistry assays of 149 HCC tissue specimens further confirmed this correlation. Taken together, the results of our study demonstrated that MSI2 correlates with EMT and has the potential to be a new predictive biomarker of HCC prognosis and invasion to help guide diagnosis and treatment of post-operative HCC patients.

## Introduction

Hepatocellular carcinoma is one of the most common highly invasive malignant tumours associated with high recurrence incidence and poor prognosis 1–2. Because of the underlying liver disease and the common resistance of HCC to existing antineoplastic agents, the efficacy of chemotherapy to HCC remains inadequate for a large number of patients 3,4. Moreover, only minimal efficacy can be achieved using radiotherapy 5 and sorafenib 6, a popular drug targeting multiple kinases. Although surgical resection is the principal therapeutic treatment for HCC patients, few patients are suitable for this treatment, and the prognosis of patients undergoing curative resection remains poor because of the high incidence of recurrence and distant metastasis 7–8. Given the high recurrence incidence, poor prognosis and the inadequate effect of conventional therapies, it is critical to identify the predictive markers of HCC invasion and prognosis to help guide diagnosis and treatment.

The MSI gene family is a recently reported gene family that was identified in stem and early progenitor cells 9–10. Two members have been identified thus far: MSI1 and MSI2. These two genes exhibit a high degree of sequence similarity 11. The literature suggests that these genes can take part in post-transcriptional regulation *via* binding to specific mRNA 12–13 and that they play a role in regulating proliferation and differentiation of stem cells of the nervous 10–12 and haematopoietic systems 9. Furthermore, emerging evidence suggests that MSI genes may play a role in cancer development. Musashi1 expression is observed in malignancy 14 and is indicative of poor prognosis in breast cancer 15, colon cancer 16–17 and gallbladder adenocarcinoma 18. Similarly, MSI2 plays a critical role in chronic myelogenous leukaemia (CML) progression 19, and high MSI2 expression predicts an unfavourable outcome in acute myeloid leukaemia (AML) 20. Nevertheless, these data are fragmentary, and thus far, the role of the MSI genes in hepatocellular carcinoma has not been explored. In this study, we profiled the expression status of both MSI1 and MSI2 in HCC, evaluated the prognostic significance of MSI1 and MSI2, and investigated the mechanism by which MSI genes affect HCC.

## Materials and methods

### Patients and specimens

Hepatocellular carcinoma specimens (tumour and matched adjacent non-tumourous tissues) were obtained from 149 consecutive patients who had undergone curative liver resection at the Affiliated Tumour Hospital of Guangzhou Medical University between June 2005 and December 2010. Ethical approval for human subjects was obtained from the research and ethics committee of the Affiliated Tumor Hospital of Guangzhou Medical University, and informed consent was obtained from each patient. The diagnoses were confirmed by histological reviews. None of the patients received anticancer treatment prior to hepatectomy, and these HCC patients were monitored after surgery until 31 September, 2012. Detailed clinicopathological parameters are listed in Table 1. The end of time to recurrence (TTR) was defined as the last date prior to evidence of metastasis or recurrence. Once evidence of HCC recurrence is confirmed, TTR will be defined as the time that recurrence was first suspected. Overall survival (OS) was computed from the day of surgery to the day of death or to the last follow-up. The 40 normal hepatic tissues used as normal controls were obtained from patients suffering from benign hepatic lesions (including hepatic haemangioma and focal nodular hyperplasia) who underwent resection.

**Table 1:** Correlation of MSI family protein expression with clinicopathological parameters

Characteristics	*n*	MSI1		*P*-value	MSI2		*P*-value
		Low	High		Low	High	
Age (years)							
≤50	79	46 (58.2%)	33 (41.8%)	0.310	36 (45.6%)	43 (54.4%)	0.190
>50	70	47 (67.1%)	23 (32.9%)		40 (57.1%)	30 (42.9%)	
AFP (μg/l)							
≤20	33	25 (75.8%)	8 (24.2%)	0.102	21 (63.6%)	12 (36.4%)	0.117
>20	116	68 (58.6%)	48 (41.4%)		55 (47.4%)	61 (52.6%)	
HBsAg							
Negative	15	11 (73.3%)	4 (26.7%)	0.414	8 (53.3%)	7 (46.7%)	1.000
Positive	134	82 (61.2%)	52 (38.8%)		68 (50.7%)	66 (49.3%)	
GGT (U/l)							
≤50	50	30 (60.0%)	20 (40.0%)	0.721	28 (56.0%)	22 (44.0%)	0.488
>50	99	63 (63.6%)	36 (36.4%)		48 (48.5%)	51 (51.5%)	
Child-Pugh score							
A	136	85 (62.5%)	51 (37.5%)	1.000	72 (52.9%)	64 (47.1%)	0.154
B	13	8 (61.5%)	5 (38.5%)		4 (30.8%)	9 (69.2%)	
Tumour size (cm)							
≤5	46	30 (65.2%)	16 (34.8%)	0.716	32 (69.6%)	14 (30.4%)	**0.003**
>5	103	63 (61.2%)	40 (38.8%)		44 (42.7%)	59 (57.3%)	
Tumour number*							
Single	92	51 (55.4%)	41 (44.6%)	**0.036**	53 (57.6%)	39 (42.4%)	**0.045**
Multiple	57	42 (73.7%)	15 (26.3%)		23 (40.4%)	34 (59.6%)	
Tumour capsule							
No/incomplete	114	72 (63.2%)	42 (36.8%)	0.842	60 (52.6%)	54 (47.4%)	0.563
Complete	35	21 (60.0%)	14 (40.0%)		16 (45.7%)	19 (54.3%)	
Tumour differentiation							
I–II	87	58 (66.7%)	29 (33.3%)	0.232	46 (52.9%)	41 (47.1%)	0.621
III–IV	62	35 (56.5%)	27 (43.5%)		30 (48.4%)	32 (51.6%)	
Vascular invasion							
No	110	71 (64.5%)	39 (35.5%)	0.442	66 (60.0%)	44 (40.0%)	**<0.001**
Yes	39	22 (56.4%)	17 (43.6%)		10 (25.6%)	29 (74.4%)	
Liver cirrhosis							
Yes	76	46 (60.5%)	30 (39.5%)	0.735	34 (44.7%)	42 (55.3%)	0.141
No	73	47 (64.4%)	26 (35.6%)		42 (57.5%)	31 (42.5%)	
BCLC stage							
0/A	82	47 (57.3%)	35 (42.7%)	**0.014**	52 (63.4%)	30 (36.6%)	**0.002**
B	32	27 (84.4%)	5 (15.6%)		14 (43.8%)	18 (56.3%)	
C	35	19 (54.3%)	16 (45.7%)		10 (28.6%)	25 (71.4%)	
Early recurrence							
No	53	32 (60.4%)	21 (39.6%)	0.726	41 (77.5%)	12 (22.6%)	**<0.0001**
Yes	96	61 (63.5%)	35 (36.5%)		35 (36.5%)	61 (63.5%)	

* Tumour number indicates number of primary tumour mass detected at the time of surgical operation.

Bold values (*P* < 0.05) are statistically significant.

### Cell culture

Three human HCC cell lines were used in this study: Huh7, SMCC7721 and MHCC-97H. All the cell lines were obtained from the Liver Cancer Institute of Fudan University (Shanghai, China) and routinely maintained in high-glucose DMEM (Life Technologies, Grand Island, NY, USA) supplemented with 10% foetal bovine serum (Life Technologies) at 37°C under 5% CO_2_.

### Immunohistochemistry assay

Formalin-fixed, paraffin-embedded tissue specimens from consenting patients were cut in 4-μm sections. The specimens were deparaffinized in xylene and rehydrated using a series of graded alcohols after being dried at 62°C for 2 hrs. The tissue slides were then treated with 3% hydrogen peroxide in methanol for 15 min. to exhaust endogenous peroxidase activity, and the antigens were retrieved in 0.01 M sodium citrate buffer (pH 6.0) using a microwave oven. After 1 hr of pre-incubation in 10% normal goat serum to prevent non-specific staining, the specimens were incubated with primary antibodies overnight at 4°C. The primary antibodies used for immunohistochemistry (IHC) assays were as follows: rabbit monoclonal antibody against MSI1 (working dilution 1:200; Epitomic, Burlingame, CA, USA), rabbit polyclonal antibodies against MSI2 (working dilution 4 μg/ml; Sigma-Aldrich, St. Louis, MO, USA) and numb homolog (NUMB; working dilution 1:100; Proteintech, Chicago, IL, USA), and mouse monoclonal antibodies against vimentin (working dilution 1:100; Santa Cruz, Dallas, TX, USA) and E-cadherin (working dilution 1:100; Abcam, Cambridge, UK). The tissue slides were treated with a non-biotin horseradish peroxidase detection system according to the manufacturer’s instructions (DAKO, Glostrup, Denmark). Two different pathologists who specialize in liver cancer evaluated the results of IHC. Both the extent and intensity of immunostaining were taken into consideration when analysing the data. The intensity of staining was scored from 0 to 3, and the extent of staining was scored from 0% to 100%. The final quantitation of each staining was obtained by multiplying the two scores. Musashi1, MSI2, E-cadherin and NUMB expression were classified as high expression if the score was higher than 1.5; if the score was 1.5 or less, the case was classified as low expression. Vimentin expression was considered high if the score reached 1.

### RNAi treatment

Briefly, MHCC-97H and SMMC7721 cells were transfected with chemically synthesized siRNA using the lipofectamine RNAiMAX transfection reagent (Life Technologies) for 48 hrs. The cells were subsequently analysed by cell invasion assays or lysed and analysed by western blot. The siRNA against MSI2 (5′-CAAUGCUGAUGUUUGAUAA-3′) was designed and chemically synthesized by Guangzhou Ribobio co. LTD (Guangzhou, China).

### Western blot

Western blots were performed as previously described 21. The primary antibodies used for western blot were as follows: mouse monoclonal antibodies against E-cadherin (BD Biosciences, San Jose, CA, USA) and vimentin (Santa Cruz); rabbit polyclonal antibodies against MSI1 (Epitomic) and MSI2 (Sigma-Aldrich); rabbit polyclonal antibodies against NUMB and α-SMA (Proteintech); rabbit polyclonal antibodies against N-cadherin (Cell Signalling Technology, Danvers, MA, USA); rabbit monoclonal antibodies against β-actin and ZO-1 (Cell Signalling Technology).

### Cell invasion assay

*In vitro* matrigel invasion assays were performed in Transwell chambers (8-μm pore size; Costar, Corning, NY, USA) according to the manufacturer’s instructions. Approximately 2 × 10^4^ cells were placed into the top chamber of each insert (BD Biosciences) and incubated at 37°C for 48 hrs. Cells that invaded through the Matrigel were stained using Hoechst 33342 (Beyotime, Jiangsu, China) and quantified.

### Immunofluoresence staining

MHCC97H and SMCC7721 cells were plated on Poly-L-Lysing coated (PLLC) slides (Thermo Corporation, Waltham, MA, USA) in 90-mm dishes at a density of 1 × 10^5^, and incubated at 37°C in 5% CO_2_ for 24 hrs. The cells on the slips were then washed with PBS three times, fixed with cold methanol for 10 min. and washed with PBST three times. After blocking with 10% BSA for 30 min., immunofluorescent staining was performed using primary antibodies against MSI2 (Rabbit monoclonal IgG, Epitomics; working dilution 1:100), E-cadherin (Mouse monoclonal IgG, BD Biosciences; working dikutin 1:100) and vimentin (Rabbit monoclonal IgG, Cell Signalling Technology; working dilution 1:200) for 2 hrs. Slides were washed in PBST three times for 5 min. Subsequently, conjugated second antibody Alexa 488 labelled anti-rabbit or mouse IgG with a dilution of 1:500 (Life Technologies) for 1 hr in the dark. Finally, the cells were counterstained and mounted with Prolong Gold antifade reagent with 4′,6-diamidino-2-phenylindole, a fluorescent nuclear dye (DAPI) (Life Technologies Corporation). The cells were then examined using a fluorescent microscope (Leica, Wetzlar, Germany) soon after counterstaining.

### Statistical analysis

All statistical analyses were performed with SPSS statistical software (version 21.0; SPSS, IBM, Armonk, NY, USA). Survival curves were constructed using the Kaplan–Meier method and analysed by the log-rank test. Significant prognostic factors identified by univariate analysis were entered into multivariate analysis using the Cox proportional hazards model. The two-tailed chi-squared test was used to analyse the association of MSI1/MSI2 expression with various clinicopathological parameters. The Student’s *t*-test was used for comparisons and the Pearson correlation test (two-tailed) was used to calculate the correlation coefficient (*r*) and *P-*value between MSI2 and vimentin staining scores, or between MSI2 and E-cadherin staining scores. Statistical significance was declared if *P* < 0.05.

## Results

### MSI1 and MSI2 expression in HCC

**Figure 1:**
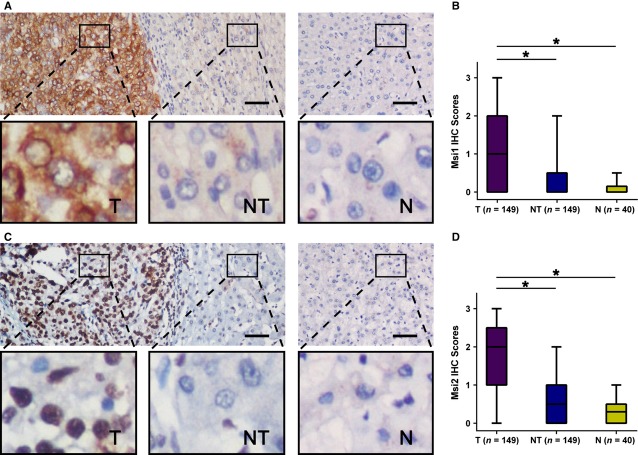
Musashi1 (MSI1) and MSI2 were significantly up-regulated in hepatocellular carcinoma (HCC). (A) Immunohistochemistry (IHC) assays of MSI1 expression in 40 normal hepatic tissues and 149 paired HCC and adjacent non-tumourous tissues. The upper left panel represents paired HCC and non-tumourous tissues and reveals high MSI1 expression in HCC tissues and low expression in adjacent non-tumourous tissues. The upper right panel represents normal hepatic tissues and reveals low MSI1 expression. Scale bars, 50 μm. Lower panels represent magnified pictures of boxed area in the corresponding upper panels. (B) MSI1 expression levels were compared among HCC, adjacent non-tumourous and normal hepatic tissue specimens. *P < 0.001. (C) IHC assays of MSI2 expression in 40 normal hepatic tissues and 149 paired HCC and adjacent non-tumourous tissues. The upper left panel represents paired HCC and non-tumourous tissues and was interpreted as high MSI2 expression in HCC tissues and low expression in adjacent non-tumourous tissues. The upper right panel represented normal hepatic tissues and was interpreted as low expression of MSI2. Scale bars, 50 μm. Lower panels represent magnified pictures of boxed area in the corresponding upper panels. (D) MSI2 expression levels were compared among HCC, adjacent non-tumourous and normal hepatic tissues. *P < 0.001.

Immunohistochemistry assays were conducted to examine the expression pattern of MSI1 and MSI2 in 149 HCC specimens (tumour and matched adjacent non-tumourous tissues) and 40 normal hepatic tissue specimens. We observed that MSI1 was primarily localized in the cytoplasm, whereas MSI2 was primarily localized in the nucleus. Western blotting of nuclear and cytoplasmic fractions further confirmed this result (Fig. S1). We detected high expression of MSI1 in 56/149 (37.6%) HCC tissues, compared with only 8/149 (5.3%) adjacent non-tumourous tissues (*P* < 0.001; Fig. 1A), whereas high expression of MSI2 was detected in 73/149 HCC tissues, compared with 12/149 adjacent non-tumourous tissues (*P* < 0.001; Fig. 1C). Musashi1 and MSI2 were both up-regulated in HCC tissues. In addition, the expression level of both MS1 (Fig. 1A) and MSI2 (Fig. 1C) was low in 40 normal hepatic tissues. These data indicated that both MSI1 (Fig. 1B) and MSI2 (Fig. 1D) expression levels were much higher in HCC tissues than that in adjacent non-tumourous and normal tissues.

### Correlation of MSI1 and MSI2 with clinicopathological parameters

To verify the functions of MSI genes, we tested the correlation of MSI1 and MSI2 expression status in 149 HCC specimens with 12 widely recognized clinicopathological parameters. Statistical analysis indicated that patients with solitary lesion or in Barcelona Clinic Liver Cancer (BCLC) stage B had a higher likelihood of high MSI1 expression (Table 1), whereas high MSI2 expression was associated with larger tumour (>5 cm in diameter), multiple tumour nodules, worse BCLC stage, vascular invasion and early recurrence (Table 1).

### Prognostic value of MSI1 and MSI2

**Figure 2:**
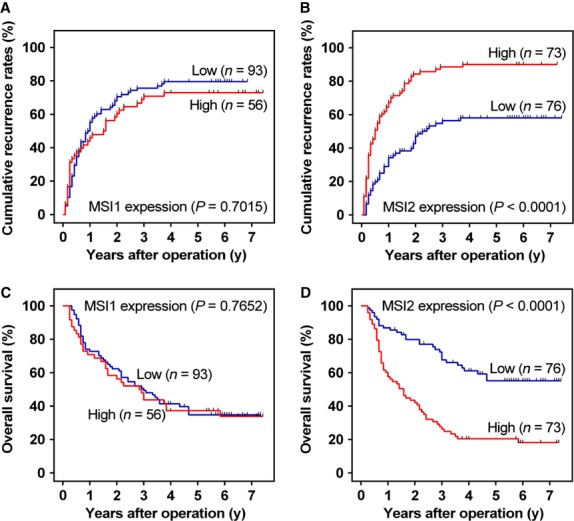
Prognostic significance assessed by Kaplan–Meier survival estimates and log-rank tests. Comparison of overall survival (OS) and time to recurrence (TTR) by Musashi1 (MSI1) (A and C) and MSI2 expression (B and D), respectively.

Furthermore, we analysed the prognostic value of MSI genes. Univariate analysis revealed that GGT level, Child-Pugh score, tumour size, tumour number, vascular invasion and BCLC stage were unfavourable predictors for OS and TTR of HCC patients, and tumour differentiation was associated with OS (Table S1). Interestingly, we found that MSI2, but not MSI1, was prognostic for TTR (*P* < 0.001) and OS (*P* < 0.0001) in HCC. The 3- and 5-year recurrence rates in the MSI2 up-regulation group were significantly higher than in the MSI2 down-regulation group (88.6% and 90.0% *versus* 54.2% and 55.9%, respectively; Fig. 2B). Similarly, the 3- and 5-year OS rates in the MSI2 up-regulation group were significantly lower than in the MSI2 down-regulation group (26.3% and 20.4% *versus* 67.7% and 55.1%, respectively; Fig. 2D). However, there was no significant difference between the high and low MSI1 expression groups (67.6% and 69.8% *versus* 71.2% and 72.7%; 44.3% and 38.3% *versus* 48.9% and 37.3%; respectively; Fig. 2A and C). Musashi2 expression status and the prognostic clinicopathological parameter found by univariate analysis (Table S1) were entered into a multivariate model to identify independent predictors of OS and TTR. Our analysis indicated that tumour size, tumour differentiation, vascular invasion and MSI2 status were independent factors that affected OS, whereas GGT, vascular invasion and MSI2 status were independent factors of TTR among HCC patients (Table 2). Among all parameters, MSI2 was the most powerful independent predictor of OS and TTR.

**Table 2:** Multivariate analysis of factors associated with OS and TTR

Variables*	OS		TTR	
	Hazard ratio (95% CI)	*P*-value	Hazard ratio (95% CI)	*P*-value
GGT (U/l) (≤50 *versus* >50)	1.509 (0.913–2.493)	0.109	1.751 (1.103–2.780)	**0.017**
Tumour size (cm) (≤5 *versus* >5)	2.161 (1.197–3.900)	**0.011**	1.538 (0.932–2.541)	0.092
Tumour differentiation (I–II *versus* III–IV)	2.092 (1.356–3.225)	**0.001**	1.227 (0.818–1.841)	0.322
Vascular invasion (no *versus* yes)	2.291 (1.433–3.663)	**0.001**	2.147 (1.373–3.359)	**0.001**
Msi2 (low *versus* high)	2.524 (1.572–4.052)	**<0.001**	2.349 (1.522–3.624)	**<0.001**

* Variables were adopted for their prognostic significance by univariate analysis (*P* < 0.05). BCLC stage was combined with several clinical indexes such as tumour size, number and tumour thrombus; we did not enter the BCLC stage into multiple analyses with these indexes to avoid any bias in analyses.

Bold values (*P* < 0.05) are statistically significant.

### siRNA knockdown of MSI2 decreases HCC cell invasion

**Figure 3:**
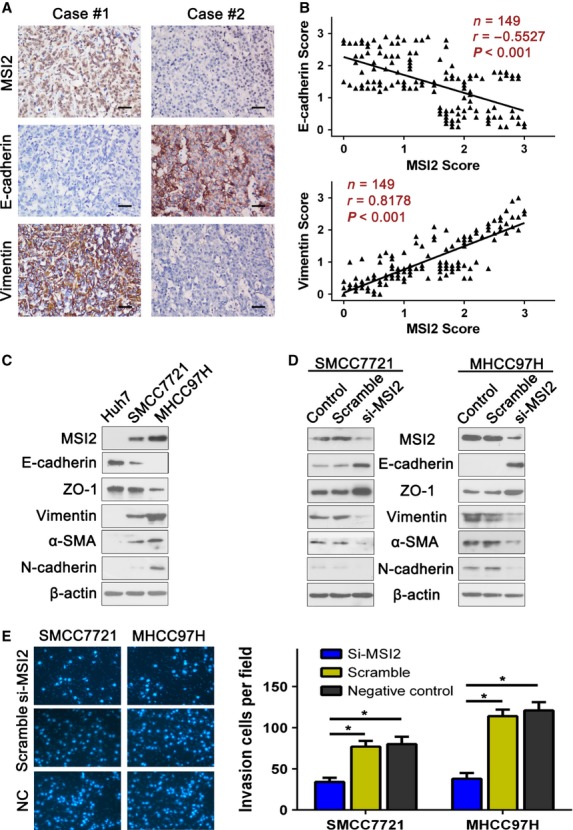
Musashi2 (MSI2) expression level correlated with the expression of epithelial–mesenchymal transition (EMT) markers, and siRNA against MSI2 significantly decreased hepatocellular carcinoma (HCC) cell invasion. (A) Serial sections of human HCC tissue were subjected to immunohistochemistry (IHC) staining with antibodies against MSI2, E-cadherin and vimentin. In case #1, high expression of MSI2 in HCC tissues was accompanied by elevated vimentin and the absence of E-cadherin. In case #2, low expression of MSI2 was accompanied by elevated E-cadherin and the absence of vimentin. Scale bars, 50 μm. (B) The two scatter plots show the correlation between MSI2 and EMT markers, E-cadherin and vimentin, in 149 HCC patients by IHC assays respectively, *P* < 0.01. MSI2 expression was positively correlated with vimentin expression and negatively correlated with E-cadherin expression. (C) The MSI2 expression pattern in HCC cell lines with different invasive potentials. MSI2 expression was positively correlated with vimentin, α-SMA, N-cadherin and negatively correlated with E-cadherin, ZO-1. (D) Transfecting siRNA against MSI2 significantly decrease MSI2 expression, up-regulated E-cadherin, ZO-1 and down-regulated vimentin, α-SMA, N-cadherin. (E) Transfecting siRNA against MSI2 significantly decreased the invasion of SMCC7721 and MHCC-97H cells, *P < 0.01.

As MSI1 expression is not correlated with the prognosis of HCC patients (Fig. 2A and C), we focused on MSI2 in subsequent analyses. Because MSI2-associated parameters were related to HCC invasion (Table 1), we next explored the correlation between MSI2 expression and HCC invasion. We chose three HCC cell lines (Huh7, SMMC7721, MHCC-97H) with different invasive capacities 22–25 and detected MSI2 expression by western blot. We could not detect MSI2 expression in Huh7, a poorly invasive HCC cell line, but MSI2 expression could be detected in SMMC7721 and MHCC-97H cells. Moreover, we observed the highest expression level of MSI2 in MHCC-97H, a well-established highly invasive HCC cell line (Fig. 3C). Next, we decreased MSI2 expression in HCC cells with siRNA knockdown of MSI2. The results of our western blot suggested that siMSI2 could significantly suppress MSI2 expression in SMMC7721 and MHCC-97H cells (Fig. 3D), and the cell invasion assay indicated that siRNA against MSI2 could significantly decrease the invasion of MHCC-97H and SMCC7721 cells (Fig. 3E). Thus, MSI2 promotes the invasion of HCC cells.

### MSI2 expression is associated with EMT

Interestingly, our western blot results also suggested that MSI2 expression was associated with the expression status of EMT markers 26 such as E-cadherin, ZO-1, vimentin, α-SMA and N-cadherin (Fig. 3C). Musashi2 expression was positively correlated with vimentin, α-SMA, N-cadherin and negatively correlated with E-cadherin, ZO-1. In addition, the relationship of MSI2 and EMT markers such as vimentin and E-cadherin was further confirmed by IHC assays in serial sections of HCC tissues (Fig. 3A and B; Table 3). Knocking down MSI2 in SMMC7721 and MHCC-97H cells resulted in lower vimentin, α-SMA, N-cadherin expression and higher E-cadherin, ZO-1 expression (Fig. 3D). Moreover, the localization of EMT markers vimentin and E-cadherin was examined by immunofluorescence. Similar to the results of western blotting, we also observed down-regulation of vimentin and up-regulation of E-cadherin when interfere MSI2 expression through siRNA (Fig. S2). These results further verified the correlation between MSI2 and EMT markers. Together, our data suggest that MSI2 may promote HCC invasion by driving EMT.

**Table 3:** Correlation of MSI2 expression with EMT markers and NUMB expression in 149 HCC tissue specimens

	Vimentin		E-cadherin		Numb	
	Low	High	Low	High	Low	High
MSI2						
Low (%)	59 (77.6)	17 (22.4)	34 (44.7)	42 (55.3)	28 (59.6)	19 (40.4)
High (%)	29 (39.7)	44 (60.3)	49 (67.1)	24 (32.9)	35 (66.0)	18 (34.0)
*P*-value	**<0.0001**		**0.008**		0.539	

Bold values (*P* < 0.05) are statistically significant.

## Discussion

Because of the high incidence of recurrence and metastasis, the long-term survival of HCC patients remains unsatisfactory. The invasion of HCC is a key factor affecting the prognosis of HCC patients. Thus, the identification of new predictive biomarkers of HCC invasion and prognosis is critical. Our study identifies MSI2 as a potential predictive biomarker of HCC invasion and prognosis. A limitation of this study is that samples were taken at hepatectomy, not biopsy. It may be more useful in clinical practice to evaluate the expression of predictive biomarkers of HCC prognosis in biopsy specimens before specific treatment such as resection. Nonetheless, based on our data, we would speculate that MSI2 staining could be a useful predictive marker in HCC biopsy samples; however this requires further study.

Musashi genes play an important role in progenitor and stem cells 12–27. Musashi1 is expressed in neural stem and progenitor cells 10–28 and helps maintain neural stem and progenitor cell populations 29. Musashi2 is expressed in neural 11 and haematopoietic 30 progenitor cells and regulates the proliferation and differentiation of progenitor cells. Musashi gene expression is correlated with the prognosis of some cancer types (breast cancer 15, colon cancer 16, gallbladder adenocarcinoma 18 and leukaemia 19,20). Nevertheless, these data are fragmentary, and the biological role of MSI genes remains elusive. To our best knowledge, we present the first data supporting the association of abnormal MSI2 expression with the invasion and prognosis of HCC. In addition, we also presented preliminary evidence for the mechanism by which MSI2 may promote HCC invasion.

We determined that MSI1 and MSI2 were abnormally expressed in HCC. Both MSI1 and MSI2 may be involved in the oncogenesis of HCC and have the potential to be biomarkers of HCC. Nevertheless, only MSI2 expression status correlated with prognosis of HCC patients. These different effects might be because of the different locations of MSI1 and MSI2 expression (Fig. S1). Byers *et al*. reported that only nuclear MSI2 levels were independently predictive of outcome in human AML 20; however, the mechanism by which MSI localization contributions to tumour outcome remains obscure. Our study suggests that MSI2 expression correlates with HCC invasion (Table 1). Musashi2-associated parameters correlated with HCC invasion, and siRNA against MSI2 significantly decreased the invasion of MHCC-97H and SMCC7721, two highly invasive HCC cell lines. In addition, we observed that MSI2 expression levels correlated with the expression status of EMT markers *in vitro*. Epithelial–mesenchymal transition is a critical event in cancer development and metastasis, and EMT can increase the invasion of cancer cells 26,31. Musashi2 expression was positively correlated with vimentin, α-SMA, N-cadherin and negatively correlated with E-cadherin, ZO-1. Additionally, the relationship of MSI2 and EMT markers, vimentin and E-cadherin, was confirmed in tissue specimens (Fig. 3). Thus, MSI2 may enhance HCC invasion by inducing EMT, thereby contributing to poor prognosis. Musashi2 is a RNA-binding protein. It has reported that MSI2 could inhibit the expression of some genes *via* binding to their mRNA 33. As our knowledge, there was no report about the mechanism of the EMT induced by MSI2. It is a hypothesis that similar RNA binding mechanism in the EMT induced by MSI2. Further studies are needed to investigate the target genes or investigate the exact mechanism. Despite all this, here, we present the first report that MSI2 expression might correlate with EMT, although more investigations are needed to confirm this conclusion. Some reports have suggested that MSI2 could inhibit the expression of NUMB 19–33, a well-accepted inhibitor of the Notch signalling pathway, thereby activating the Notch signal pathway 34,35. Therefore, we detected the expression level of NUMB in HCC tissue specimens by IHC (Fig. S3). Our analysis suggested that deficiency of NUMB expression correlated with poor prognosis (Fig. S4), although there was no correlation between MSI2 and NUMB in HCC (Table 3). This result demonstrated that deficiency of NUMB might take part in the development of hepatocellular carcinoma, but MSI2 acts in a NUMB-independent manner to promote HCC.

In conclusion, our study revealed that both MSI1 and MSI2 are abnormally expressed in hepatocellular carcinoma, but only the abnormal expression of MSI2 was associated with poor prognosis in a large series of hepatocellular carcinoma. Musashi2 may contribute to HCC invasion by inducing EMT but is not correlated with NUMB expression. Although the mechanism remains to be elucidated, MSI2 has the potential to be a predictive marker of HCC invasion and prognosis.

## Supporting information

Figure S1 Location of MSI1 and MSI2 in HCC. (A) IHC assay showed that MSI1 was primarily localized in the cytoplasm, MSI2 was primarily localized in the nucleus. (B) Western blot indicated that MSI1 could only be detected in the cytoplasmic extraction, while MSI2 could only be detected in the nuclear extraction.

Figure S2 Immunofluorescent stainings for MSI2, vimentin and Ecadherin. Immunofluorescent stainings for MSI2 (red), E-cadherin (green) and Vimentin (red) were performed on hepatocellular carcinoma MHCC97H and SMCC7721 cells that treated with si-MSI2 (original magnification: 400-fold).

Figure S3 NUMB was significantly down-regulated in HCC. IHC assays of NUMB expression in 100 paired HCC and adjacent non-tumourous tissues indicated that NUMB was significantly down-regulated in HCC tissues, compared with adjacent non-tumourous tissues. The upper left panel represented paired HCC and non-tumourous tissues and was interpreted as low expression of NUMB in HCC tissues, high expression in adjacent non-tumourous tissues. The right panel was the box plot graph showing NUMB expression level compared among HCC, adjacent non-tumourous and normal hepatic tissues. ^*^*P* < 0.001.

Figure S4 Prognostic significance assessed by Kaplan–Meier survival estimates and log-rank tests. Comparison of OS (overall survival) and TTR (time to recurrence) by NUMB (**A** and **B**).

Table S1 Univariate analysis of factors associated with survival and recurrence.
