# Supplementary figures and images for: Musashi2 predicts poor prognosis and invasion in hepatocellular carcinoma by driving epithelial–mesenchymal transition
Source: J Cell Mol Med. 2013 Oct 31;18(1):49–58. doi: 10.1111/jcmm.12158 (PMC3916117; doi:10.1111/jcmm.12158)

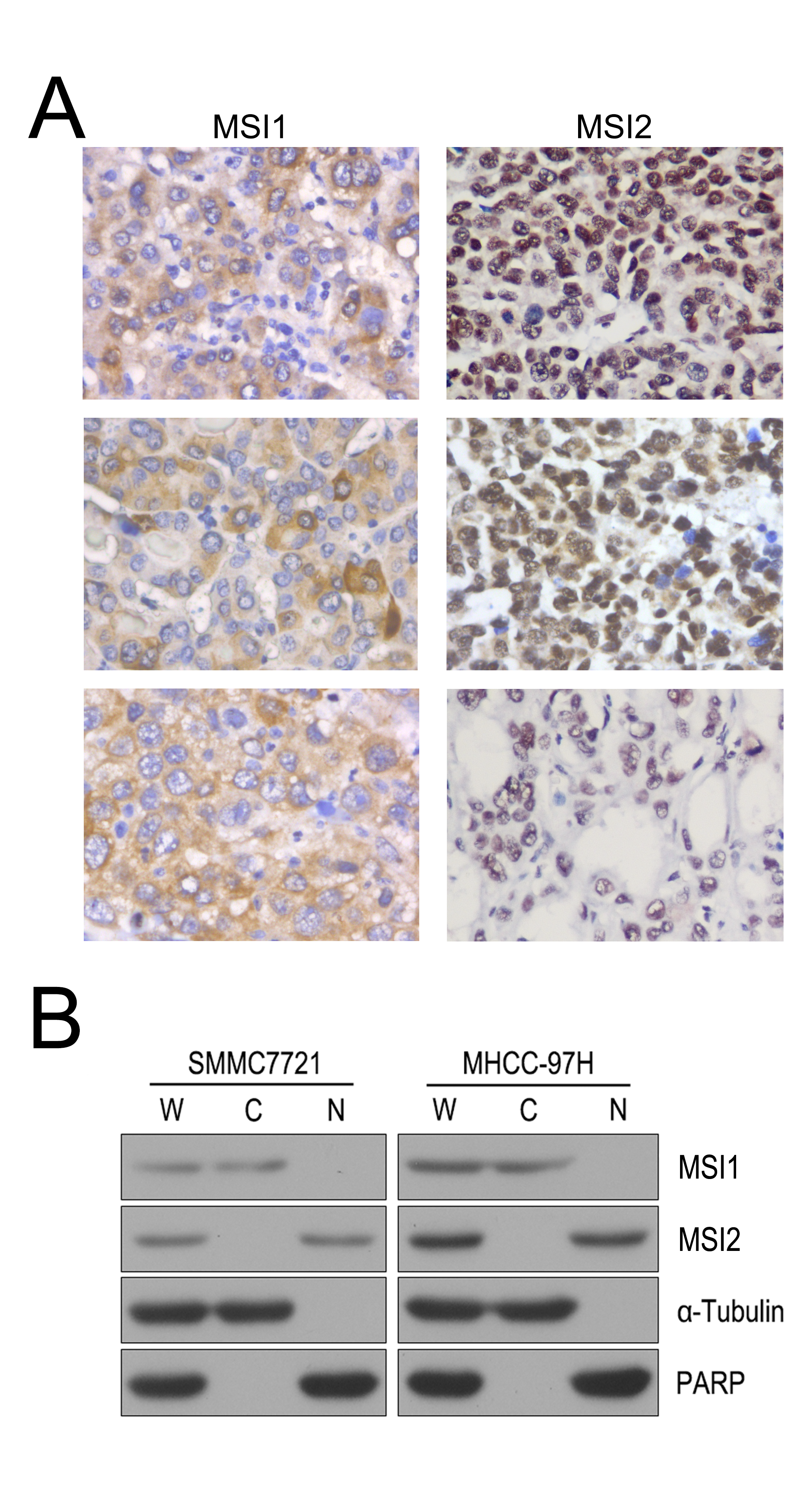

Supplement: Figure S1 — Location of MSI1 and MSI2 in HCC. (A) IHC assay showed that MSI1 was primarily localized in the cytoplasm, MSI2 was primarily localized in the nucleus. (B) Western blot indicated that MSI1 could only be detected in the cytoplasmic extraction, while MSI2 could only be detected in the nuclear extraction. [file jcmm0018-0049-sd1.tif]

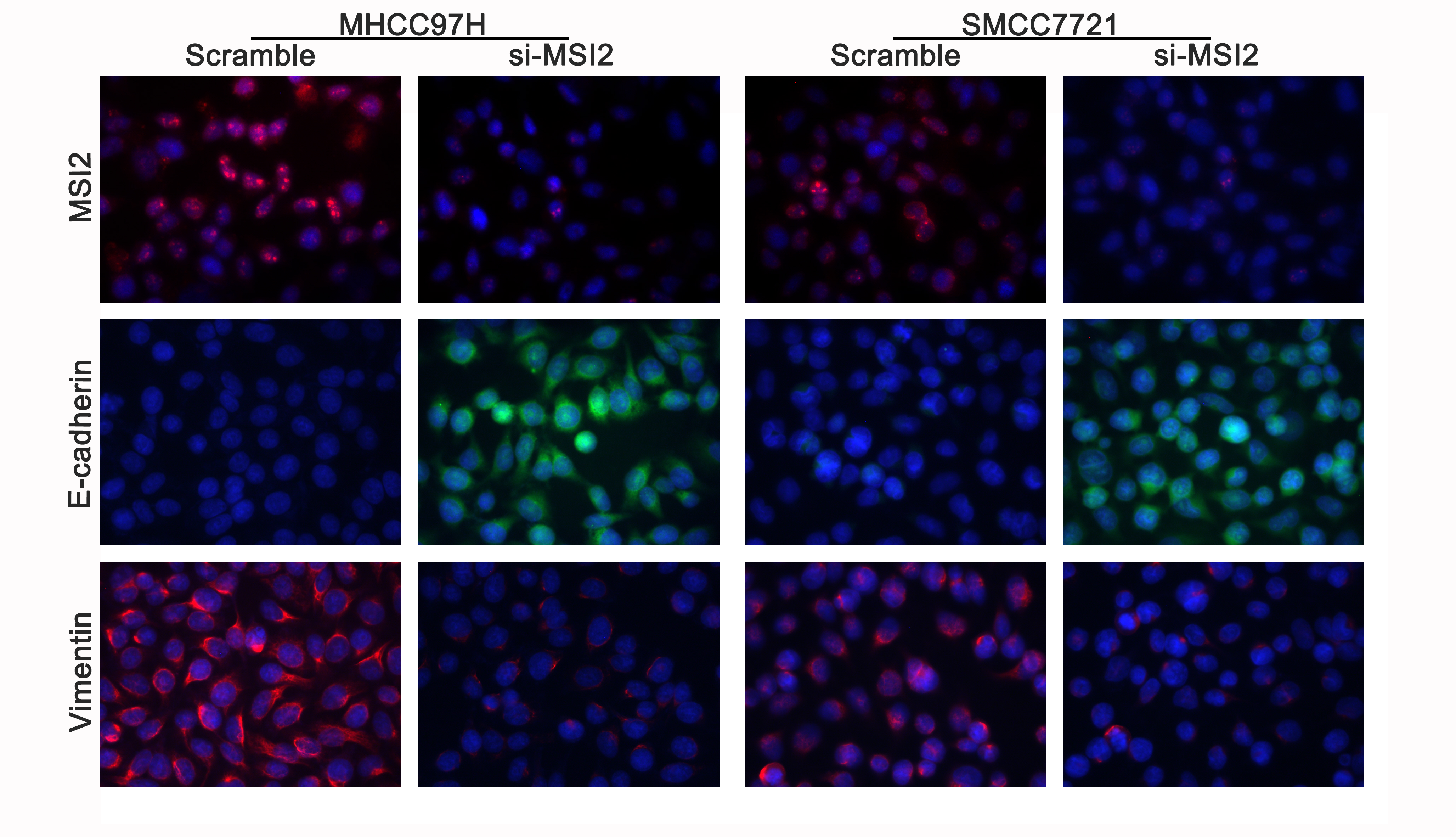

Supplement: Figure S2 — Immunofluorescent stainings for MSI2, vimentin and Ecadherin. Immunofluorescent stainings for MSI2 (red), E-cadherin (green) and Vimentin (red) were performed on hepatocellular carcinoma MHCC97H and SMCC7721 cells that treated with si-MSI2 (original magnification: 400-fold). [file jcmm0018-0049-sd2.tif]

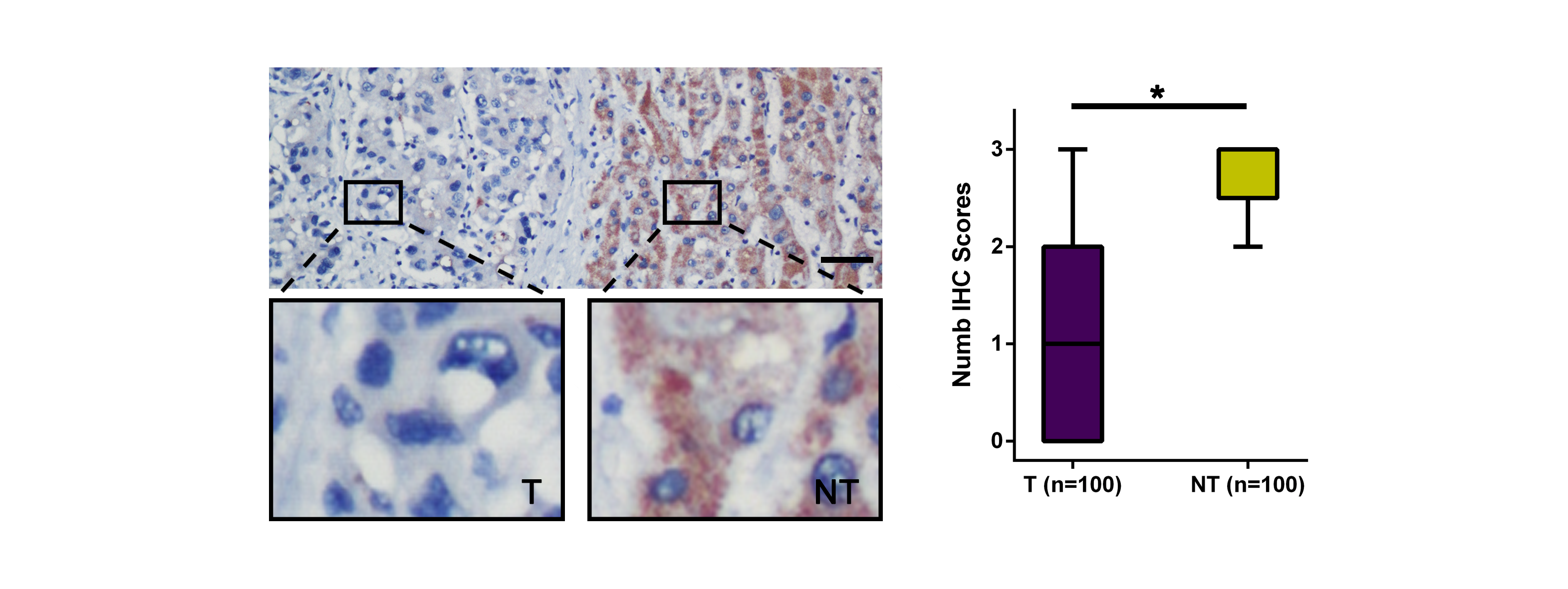

Supplement: Figure S3 — NUMB was significantly down-regulated in HCC. IHC assays of NUMB expression in 100 paired HCC and adjacent non-tumourous tissues indicated that NUMB was significantly down-regulated in HCC tissues, compared with adjacent non-tumourous tissues. The upper left panel represented paired HCC and non-tumourous tissues and was interpreted as low expression of NUMB in HCC tissues, high expression in adjacent non-tumourous tissues. The right panel was the box plot graph showing NUMB expression level compared among HCC, adjacent non-tumourous and normal hepatic tissues. *P < 0.001. [file jcmm0018-0049-sd3.tif]

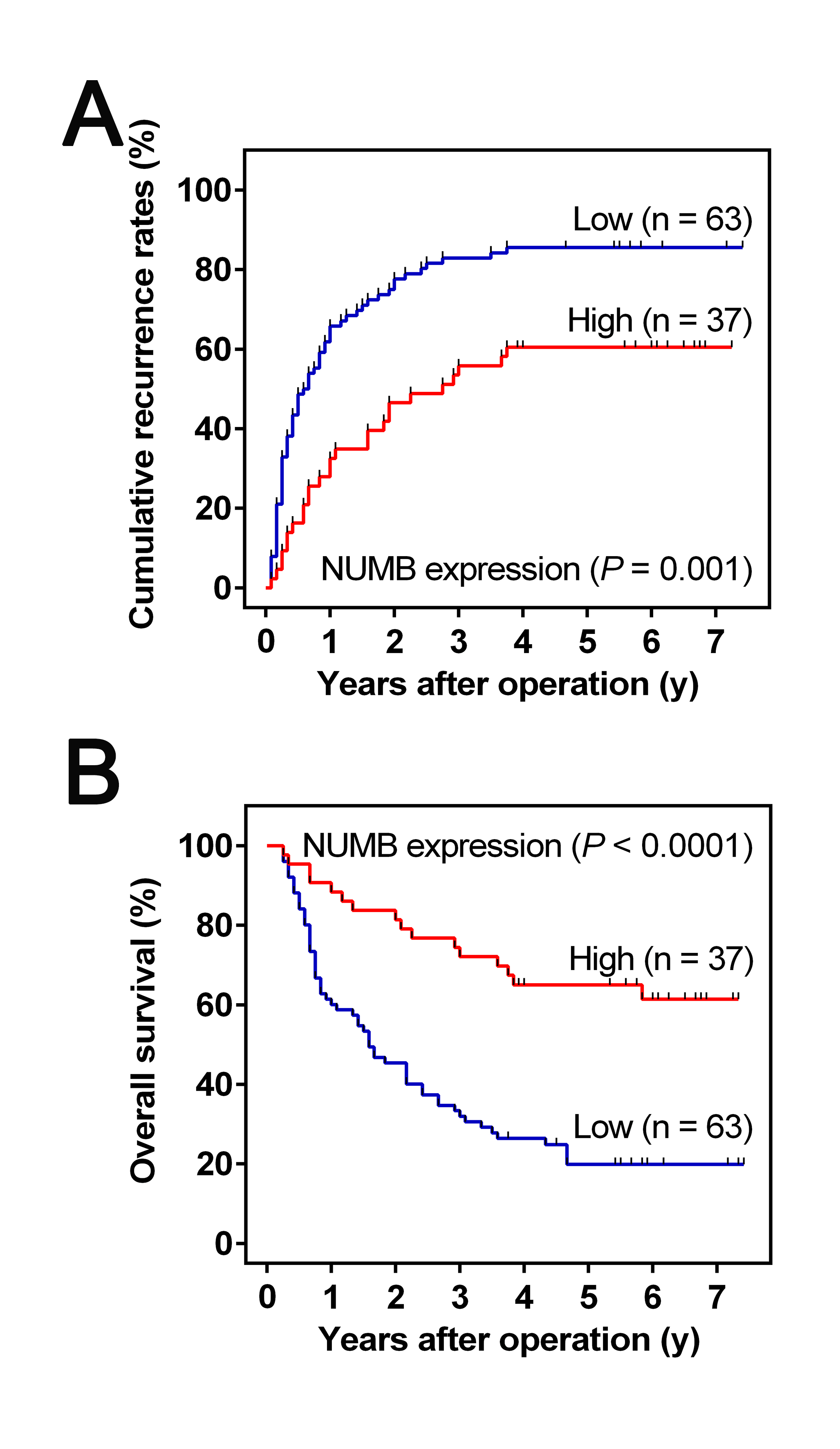

Supplement: Figure S4 — Prognostic significance assessed by Kaplan–Meier survival estimates and log-rank tests. Comparison of OS (overall survival) and TTR (time to recurrence) by NUMB (A and B). [file jcmm0018-0049-sd4.tif]
