# Supplementary material for: Musashi2 predicts poor prognosis and invasion in hepatocellular carcinoma by driving epithelial–mesenchymal transition
Source: J Cell Mol Med. 2013 Oct 31;18(1):49–58. doi: 10.1111/jcmm.12158 (PMC3916117; doi:10.1111/jcmm.12158)
Supplement: Table S1 — Univariate analysis of factors associated with survival and recurrence. [file jcmm0018-0049-sd5.doc]

Supplemental Table 1. Univariate analysis of factors associated with survival and recurrence

| Variables | OS | |  | TTR | |
| --- | --- | --- | --- | --- | --- |
|  | Hazard ratio (95%CI) | *P* value |  | Hazard ratio (95%CI) | *P* value |
| Age, years (≤50 *vs.* >50) | 0.784 (0.519-1.183) | 0.246 |  | 0.859 (0.579-1.275) | 0.451 |
| AFP (μg/l) (≤20 *vs.* >20) | 1.739 (0.999-3.028) | **0.050** |  | 1.643 (0.973-2.774) | 0.063 |
| HBsAg (negative *vs.* positive) | 0.493 (0.215-1.128) | 0.094 |  | 0.814 (0.423-1.565) | 0.537 |
| GGT (U/l) (≤50 *vs.* >50) | 1.928 (1.202-3.093) | **0.006** |  | 1.921 (1.230-3.001) | **0.004** |
| Child-Pugh score (A *vs.* B) | 2.064 (1.168-3.647) | **0.013** |  | 1.993 (1.060-3.749) | **0.032** |
| Tumor size (cm) (≤5 *vs.* >5) | 2.898 (1.747-4.807) | **<0.0001** |  | 2.222 (1.390-3.554) | **0.001** |
| Tumor number (single *vs.* multiple) | 1.964 (1.301-2.963) | **0.001** |  | 1.798 (1.205-2.683) | **0.004** |
| Tumor capsule (no/incomplete *vs.* complete) | 1.347 (0.821-2.211) | 0.238 |  | 1.221 (0.760-1.962) | 0.409 |
| Tumor differentiation (I-II *vs.* III-IV) | 1.905 (1.266-2.865) | **0.002** |  | 1.331 (0.894-1.982) | 0.159 |
| Vascular invasion (no *vs.* yes) | 3.071 (1.991-4.739) | **<0.0001** |  | 3.072 (2.006-4.705) | **<0.0001** |
| Liver cirrhosis (no *vs.* yes) | 0.692 (0.454-1.056) | 0.088 |  | 0.700 (0.487-1.0442) | 0.079 |
| BCLC stage (0/A vs. B *vs.* C) | 1.930 (1.525-2.442) | **<0.0001** |  | 2.007 (1.593-2.528) | **<0.0001** |
| MSI1 (low *vs.* high) | 1.070 (0.682-1.678) | 0.768 |  | 0.925 (0.615-1.391) | 0.708 |
| MSI2 (low *vs.* high) | 3.160 (2.023-4.937) | **<0.0001** |  | 2.791 (1.852-4.288) | **<0.0001** |
